# External quality assessment for molecular typing of *Salmonella* 2013–2015: performance of the European national public health reference laboratories

**DOI:** 10.1007/s10096-017-3015-7

**Published:** 2017-06-01

**Authors:** M. B. F. Jensen, S. Schjørring, J. T. Björkman, M. Torpdahl, E. Litrup, E. M. Nielsen, T. Niskanen

**Affiliations:** 1 Unit of Foodborne Infections, Statens Serum Institut, 2300 Copenhagen, Artillerivej 5 Denmark; 2 European Programme for Public Health Microbiology Training, European Centre for Disease Prevention and Control (ECDC), Granits väg 8, 171 65 Solna, Stockholm Sweden; 3 European Centre for Disease Prevention and Control (ECDC), Granits väg 8, 171 65 Solna, Stockholm Sweden

## Abstract

We report the results of three consecutive External Quality Assessments (EQAs) for molecular subtyping of *Salmonella* to assess the performance of the European national public health reference laboratories (NPHRLs). The EQA included the molecular typing methods used for European enhanced surveillance of human *Salmonella* infections: pulsed field gel electrophoresis (PFGE), including gel analysis by the use of the software BioNumerics, and 5-locus multiple locus variable number of tandem repeat analysis (MLVA) for serovar Typhimurium. The participation in the PFGE laboratory part was higher (27/35) than in the gel analysis (19/35) and MLVA (15/35), suggestive of the need for capacity building in methods requiring specialized equipment (MLVA) or software (gel analysis). The majority (25/27) of the participating NPHRLs produced inter-laboratory comparable PFGE gel(s). Two laboratories continued to produce low-quality gels and should have additional technical assistance in the future. In particular, two gel quality evaluation parameters, measuring “image acquisition and running conditions” and “bands”, were identified to cause gel quality problems throughout the EQAs. Despite the high number of laboratories participating in the PFGE laboratory part, the participation in gel analysis was low, although increasing. In the MLVA part, the NPHRLs correctly assigned 96% (405/420) allelic profiles according to the nomenclature. In conclusion, the EQAs identified critical parameters for unsuccessful performance and helped to offer assistance to those laboratories that needed it most. The assessments supported the development of quality in molecular typing and promoted the harmonization of subtyping methods used for EU/EEA-wide surveillance of human *Salmonella* infections.

## Introduction

Salmonellosis is one of the most frequent zoonotic diseases worldwide. In Europe, *Salmonella enterica* ssp. *enterica* continues to be the second most reported zoonosis and a common cause of foodborne outbreaks responsible for about 20% of all outbreaks in the EU in 2014 [1]. Symptoms range from self-limiting diarrhoea to life-threatening sepsis. Molecular typing of *Salmonella* strains aids in improving the identification, investigation, and control of an outbreak source so that prevention of further spread is effective. In the EU, the European Centre for Disease Prevention and Control’s (ECDC’s) disease programme for Food- and Waterborne Diseases and Zoonoses (FWD DP) is responsible for the surveillance of salmonellosis. In order to improve the surveillance of foodborne infections, molecular typing data have been reported to the European Surveillance System (TESSy) by the Member States since 2012 [2]. For *Salmonella*, this included pulsed field gel electrophoresis (PFGE) data for all serovars and multiple locus variable number of tandem repeat analysis (MLVA) data for *Salmonella* Typhimurium. The molecular typing for surveillance is based on the capacity of the laboratories in the European FWD network (FWD-Net) to produce typing data of good quality to allow for reliable cross-border surveillance and outbreak detection. The current molecular typing methods used for EU-wide surveillance and outbreak investigation of *Salmonella*, are PFGE suitable for typing all serovars [3, 4], and MLVA for *S*. Typhimurium [5, 6] and *S*. Enteritidis [7], two of the most prevalent serovars in the EU/EEA [1]. MLVA provides a higher discriminatory power compared to PFGE within *S*. Typhimurium, and has been used for surveillance and outbreak investigations in the past decade [8–10]. Likewise, there was a need for a more discriminatory method than PFGE for detecting outbreaks of serovar Enteritidis. Therefore, a multi-laboratory validation study of a 5-locus MLVA scheme for *S*. Enteritidis was recently carried out [7] and since June 2016 MLVA data for *S*. Enteritidis have also been included in TESSy. Thus, MLVA for *S*. Enteritidis will be included in future EQAs. Protocols for PFGE and both MLVA methods have been standardized to produce comparable results across laboratories [7, 11–15].

Since 2012, the National Public Health Reference Laboratories (NPHRLs) in the FWD-Net have been given the opportunity to participate in External Quality Assessments (EQAs) for the molecular typing methods used in the European surveillance of foodborne pathogens. The evaluation of the PFGE results of the first round of EQAs (2012-EQA) for the three pathogens *Salmonella*, *Listeria*, and verotoxin-producing *E. coli* is reported by Schjørring et al. 2016 [16]. In the present study, the results of the following three EQA rounds related to molecular typing of *Salmonella* strains by PFGE for all serovars and MLVA for *S*. Typhimurium will be presented. The aims of each EQA round were to assess the quality of PFGE typing and the comparability of the test results among the NPHRLs in the FWD-Net, as well as to determine and ensure the quality and integrity of the *S*. Typhimurium MLVA results among the participating laboratories. Here, we combined the results of the three rounds which took place in the years 2013–15 and assessed the development in the capability of the NPHRLs in producing molecular typing data that can be used for supporting the surveillance of *Salmonella* infections in Europe. We will focus on development in the capacity to perform the standard methods, the quality and comparability of the typing data, and the identification of common quality issues.

## Material and methods

### Participants

The EQAs were open to one NPHRL in each of the 30 FWD-Net countries in the EU/EEA and five EU candidate countries. The laboratories could choose to perform all or selected method(s) of the EQA. Over the 3-year period, the number of participating countries increased from 24 to 27. Twenty-one (60%) NPHRL participated in all three EQA rounds. These studies were coordinated by the Unit for Foodborne Infections, Statens Serum Institut (SSI), Copenhagen, Denmark in accordance with the International Standard ISO/IEC 17043:2010 [17]. Technical reports of the individual EQA distributions have been published by ECDC [18–20].

### Test strains

Clinical isolates from the SSI strain collection were selected for inclusion. For each EQA round, candidate strains were selected to represent serovars relevant for the epidemiological situation in Europe, including recent outbreak strains (Table 1). For MLVA, *S*. Typhimurium strains were selected to cover common allelic profiles, and include three strains (a-c) repeated in all EQAs (Table 2). Stability testing and preparation of strains for distribution were performed according to the International Standard ISO/IEC 17043:2010 [17]. The final selection of ten test strains for each PFGE and MLVA testing was made from the candidate strains that remained stable during ten consecutive passages. Repeat strain c had generated a stable one-repeat change in the STTR10-locus during storage at SSI (changed from 23 to 24 repeats, Table 2). Replicates of each isolate were prepared on agar stabs (SSI Diagnostica, Hillerød, Denmark), blind coded and dispatched by courier. All packages were delivered within 2 weeks (mean 2 days). No damage to the shipments was reported. The coordinating centre provided, both within the packages and individually by email, standard protocols and individual letters stating the unique blind-coded strain IDs, and detailed instructions on setting up the database, Image Acquisition recommendations, obtaining correct MLVA results, importing MLVA into BioNumerics (Applied Math, Sint-Marten-Laten, Belgium) and exporting data from BioNumerics. In addition, pre-configured BioNumerics databases (for all versions of the software), Excel templates for allele-calling and calibration of raw fragment sizes to verified sizes of the reference strains [21] were made available for the participants.

**Table 1:** *Salmonella* serovars included for PFGE typing in each *Salmonella* EQA round, 2013–2015

EQA	Test strain	*Salmonella* serovar
2013	1–10	Enteritidis (two strains), Infantis^a^, Kentucky, Mbandaka, Montevideo, O:4,5,12;H:i:-, Poona, Stanley^b^, and Strathcona
2014	1–10	Agona, Enteritidis, Hadar, Infantis^a^, Manhattan, Mikawasima, O:4,5,12;H:i:-, Poona, Stanley^b^, and Typhimurium
2015	1–10	Chester, Enteritidis, Infantis^a^, Java, Javiana, O:4,5,12;H:i:-, Poona, Reading, Stanley^b^, and Typhimurium

Repeat strains (a and b) included in all EQA rounds

**Table 2:** Allelic profiles included for MLVA of *Salmonella* Typhimurium in each *Salmonella* EQA round, 2013–2015

EQA	Test strain	MLVA				
		Allelic profile				
		STTR9	STTR5	STTR6	STTR10	STTR3
2013	11	2	15	7	10	212
	12	4	20	7	7	212
	13	2	20	13	12	12
	14^a^	3	13	NA	NA	211
	15	3	14	9	NA	211
	16^b^	3	12	9	NA	211
	17	3	18	11	NA	211
	18	3	15	NA	NA	111
	19	3	14	11	3	309
	20^c^	3	16	15	23*	311
2014	11	2	11	8	9	212
	12	2	13	3	NA	212
	13	2	20	9	7	212
	14	3	10	10	NA	211
	15^b^	3	12	9	NA	211
	16^a^	3	13	NA	NA	211
	17	3	13	9	NA	211
	18^c^	3	16	15	23*	311
	19	3	18	11	NA	211
	20	4	16	9	9	211
2015	11^b^	3	12	9	NA	211
	12	5	19	9	11	211
	13	2	15	7	9	212
	14	4	17	13	9	111
	15^a^	3	13	NA	NA	211
	16	3	11	11	NA	211
	17	8	14	NA	NA	211
	18^c^	3	16	15	24*	311
	19	3	12	6	NA	211
	20	3	18	14	15	311

Repeat strain (a–c) included in all EQA rounds. Repeat strain c had gained a repeat in the STTR10 locus before distribution in the 2015-EQA

*NA* not applicable (locus not present) [12]

*Single locus variants (﻿SLV) of the repeat strain c in STTR10

### Reference strains

The participants could request the PFGE reference size marker *S*. Branderup H9812 and the 33 *S. *Typhimurium MLVA reference strains, used for normalization of measured fragment size, including STm-SSI032 (3,17,21,18,311) and STm-SSI033 (2,13,9,11,112) recently added to the original MLVA reference set [11, 21].

### Typing analysis

Participants were instructed to use the Standard PulseNet PFGE protocol for *Salmonella* [15], and analyse their PFGE gel using the pre-configured BioNumerics database generating comparable profiles by normalization to the size standard and band assignment. For allelic profiling, the 5-locus *S*. Typhimurium MLVA standard protocol was suggested including the agreed nomenclature [12, 14]. Normalization of measured fragment size to actual size could be performed according to the verified fragment size of the 33 reference strains using the distributed calibration file [21]. The allele-calling Excel file returned the true allele number when a submitted fragment size matched to a predesignated allele number. This nomenclature provides a string of five digits representing the true number of repeat units in each of the five loci. An absent allele is reported as NA [12].

### Reporting

PFGE gel images were submitted to the coordinating centre in a tagged image file format (TIFF file) and by returning an online questionnaire indicating the position on the gel of each coded test strain. The participants who also performed the subsequent gel analysis in BioNumerics submitted an export file including all results and additional information. MLVA results were submitted as allelic profiles, either submitted via the online questionnaire for each coded test strain, or submitted as part of the export file from BioNumerics. The coordinators imported the results to a dedicated BioNumerics database, and reported to the participants if errors in the submission process were identified.

### Scoring of participant results

Analysis of submitted results was undertaken by the coordinating centre, and individual evaluation and feedback reports were provided to each participant summarizing, scoring, and commenting on the results. Areas for improvement were identified and optimization suggested. In addition, certificates of participation were provided to the laboratories. Participants were given an anonymised and confidential laboratory number.

#### PFGE gel quality

The quality of the raw PFGE gels (laboratory part) was graded according to the modified ECDC FWD MolSurv Pilot - SOPs 1.0, PulseNet US protocol PFGE Image Quality Assessment (TIFF Quality Grading Guidelines) [16, 18–20]. The evaluation examines seven gel parameters: (1) image acquisition and running conditions (TIFF file and band spacing of global standard), (2) cell suspension (evenness of DNA concentrations), (3) bands (appearance of bands, distint/distorted/fuzzy), (4) lanes (straightness), (5) restriction (completeness), (6) gel background (debris or clear), and (7) DNA degradation (smearing), using four score values 1–4. An acceptable gel quality (score of 2 or better) should be achieved in each parameter. A non-accepted low quality score of 1 in just one parameter impacts the ability to further analyse the image and produce comparable profiles across laboratories. Results are presented as accepted (1)/non-accepted (0), together with the total percentage score of the maximal of 28 points.

#### PFGE gel analysis

Participants with access to the specialized software BioNumerics were asked to analyse the PFGE gel produced in their own laboratory. The performance of this gel analysis was graded according to the BioNumerics Gel Quality Grading Guidelines, developed by the coordinating centre [16, 18–20]. The grading examined five parameters: (1) position of gel, (2) strips, (3) curves, (4) normalization, and (5) band assignment. Each parameter with scores ranging from 1 to 3. As for the gel scoring, acceptable scores of 2 or better should be achieved in each parameter. The parameter “band assignment” was graded according to the quality of the gel, i.e., a laboratory producing a non-accepted gel could still achieve an accepted score in the following gel analysis. Results are presented as the total percentage score of the maximal of 15 points.

#### MLVA

The allelic profile of each test strain was scored as correct (“1”) or incorrect (“0”), and the percentage of correctly assigned allelic profiles generated a total score from zero to 100% correct profiles.

One-repeat single locus variants (SLVs) of the highly variable loci, STTR5, STTR6, and STTR10 were accepted when evaluating the results. Changes in the rapidly changing loci can be unavoidable during passage and transport.

## Results

### Participation

The participation rate was highest among the FWD-Net countries, with 26 of the 30 countries (87%) participating in at least one of the three EQA rounds. The participation rate was 60% (3/5) among the EU candidate countries. The number of participants increased from 24 (69%) in the 2013-EQA to 27 (77%) in the 2015-EQA (Table 3). In each EQA, PFGE received more participations (*n* = 22–25) than MLVA typing (*n* = 14, Table 3). Due to major technical problems, the PFGE gel from one of two new participants (2015-EQA) was un-analysable and excluded from the results presented. Additional laboratories signed up for the EQAs, but refrained to submit the results: two in the PFGE and three in the MLVA part in the 2013-EQA, and two in the PFGE part of the 2014-EQA. Only participants submitting analysable results are presented.

**Table 3:** Number of laboratories submitting results to the *Salmonella* EQA rounds by method and year, 2013–2015

EQA	PFGE, *n* (%)			MLVA, *n* (%)
	Gel	Gel and analysis	Total (%)	
2013 (*n* = 24)	6	16	22 (92)	14 (58)
2014 (*n* = 26)	8	17	25 (96)	14 (54)
2015 (*n* = 27)	8*	17	25 (93)	14 (54)

*Gel* Submission of PFGE gel image

*Gel and analysis *﻿Submission of PFGE gel image including profile analysis in BioNumerics

*One laboratory submitted an un-analysable PFGE gel due to major technical problems, and was excluded from analyses, thus *n* = 7 evaluated results

### PFGE gel quality

Most of the participating NPHRLs (27/29) performed PFGE typing in at least one EQA during the 3-year period (Table 4). Applying acceptable versus non-acceptable performance as means of performance, more than half of the participants (15/27) displayed a high performance level, i.e., generating acceptable gels on each submission (Table 4). Four (4/27) laboratories (no. 130, 144, 129, 138) showed a deteriorating level of performance. These laboratories provided acceptable gel(s) in one or two of the early distributions; however, they produced non-acceptable gels in later EQAs (Table 4). Conversely, four participants (no. 114, 140, 148, 145) improved their performance by demonstrating their ability to generate acceptable gels after initially producing non-acceptable gels in the earlier EQA rounds (Table 4). Two laboratories (no. 132 and 160) were unable to produce an acceptable gel; laboratory no. 132 in all EQAs, and no. 160 in EQA-2014 and EQA-2015 (Table 4). In the two rounds (2014-EQA and 2015-EQA), laboratory no. 160 did not use the recommended PFGE protocol for *Salmonella*, and subsequently generated very fuzzy bands. Laboratory no. 132 had various problems, the main ones being overexposure of the gel image and not using the correct PFGE protocol/running conditions. A common feature in the two laboratories was the inability to produce a gel that could be normalized or show distinguishable bands, thereby obtaining the lowest score (non-acceptable) in the two parameters “image acquisition and running conditions” and “bands”. In general, non-acceptable scores were given to at least two laboratories for these two parameters in each of the three EQA rounds, while no non-acceptable scores were given in three parameters (cell suspension, lanes and gel background). In the remaning two parameters (restriction and DNA degradation), non-acceptable scores were only given in 2014 (Table 5). Among the 71 gels evaluated, only five (7%) gels (three laboratories) obtained a 100% total score (Table 4), reflecting the difficulty in producing an exemplary gel with respect to all evaluation parameters.

**Table 4:** Individual laboratory PFGE gel quality result (acceptable or non-acceptable) in *Salmonella* EQA rounds by year, 2013–2015

Number of EQAs (no. of participants)	Laboratory number	Performance level			Total percentage score^a^		
		2013	2014	2015	2013	2014	2015
3 (*n* = 20)	92	1	1	1	100	100	93
	147	1	1	1	100	96	100
	19	1	1	1	96	100	82
	36	1	1	1	93	96	96
	55	1	1	1	93	86	86
	106	1	1	1	89	96	75
	49	1	1	1	86	89	86
	134	1	1	1	82	96	82
	100	1	1	1	82	89	86
	77	1	1	1	71	89	75
	128	1	0	1	79	82	89
	142	1	0	1	71	64	89
	130	1	1	0	79	82	64
	144	1	1	0	79	79	68
	129	1	1	0	75	93	86
	138	1	0	0	86	64	71
	114	0	1	1	82	79	79
	140	0	1	1	82	93	93
	148	0	0	1	64	54	75
	132	0	0	0	75	64	68
2 (*n* = 4)	150	1	1	–	82	75	–
	108	–	1	1	–	79	86
	145	–	0	1	–	71	71
	160	–	0	0	–	79	64
1 (*n* = 3)	406	1	–	–	79	–	–
	125	–	1	–	–	75	–
	180	–	–	1	–	–	89

*1* acceptable gel, *0* non-acceptable gel, – no participation

^a^Total percentage score of the maximum score of 28 points

**Table 5:** Number of non-acceptable PFGE gel scores and average score in *Salmonella* EQA rounds by parameter and year, 2013–2015

Parameter	Condition of excellent score [4]	No. of non-acceptable gels (%)			Average score		
		2013 *n* = 22	2014 *n* = 25	2015 *n* = 24	2013	2014	2015
Image acquisition and running conditions	Wells included, bottom band 1.5 cm from edge, and spacing of standard match global standard	2 (9%)	2 (8%)	3 (13%)	2.4	3.0	2.9
Cell suspension	Even distribution of DNA	0	0	0	3.9	4.0	3.6
Bands	Clear and distinct bands	2 (9%)	7 (28%)	5 (21%)	2.5	2.5	2.5
Lanes	Straight lanes	0	0	0	3.8	3.7	3.6
Restriction	Complete restriction in all lanes	0	2 (8%)	0	3.8	3.7	3.8
Gel background	Clear background	0	0	0	3.3	2.9	3.3
DNA degradation	No degradation	0	2 (8%)	0	3.5	3.5	3.1

Scores given according to the TIFF Quality Grading Guidelines [18–20]. Score of 1: non-acceptable, scores of 2, 3 or 4: acceptable

### PFGE gel analysis

Nineteen (19/27, 70%) of the NPHRLs performing PFGE analysed their PFGE profiles by the use of BioNumerics and demonstrated a high-quality performance level (Table 6). Fifteen (79%) laboratories performed acceptable gel analysis in every distribution they took part in. Two participants (no. 128 and 129) improved their performance by producing acceptable gel analysis after producing non-acceptable analysis in earlier EQA rounds. In the 2015-EQA, all participants produced an acceptable gel analysis. Among the 40 gel analyses evaluated in total, 12 (30%) gel analyses obtained a 100% score (Table 6).

**Table 6:** Individual laboratory gel analysis quality result (acceptable or non-acceptable) in *Salmonella* EQA rounds by year, 2013–2015

Number of EQAs (no. of participants)	Laboratory number	Performance level			Total percentage score^a^		
		2013	2014	2015	2013	2014	2015
3 (*n* = 14)	19	1	1	1	100	100	80
	147	1	1	1	100	93	93
	36	1	1	1	100	93	93
	49	1	1	1	100	93	93
	77	1	1	1	100	93	93
	92	1	1	1	100	87	87
	142	1	1	1	93	93	93
	134	1	1	1	93	93	93
	106	1	1	1	93	87	100
	55	1	1	1	87	93	93
	148	1	0	1	87	53	73
	130	1	0	1	80	67	73
	129	0	1	1	87	93	93
	128	0	0	1	73	67	100
2 (*n* = 3)	150	1	1	*–*	93	87	*–*
	100	*–*	1	1	*–*	100	100
	108	*–*	1	1	*–*	100	87
1 (*n* = 2)	406	1	*–*	*–*	87	*–*	*–*
	132	*–*	*–*	1	*–*	*–*	93

Scores given according to the BioNumerics Gel Quality Grading Guidelines, [18–20]. *1* acceptable gel, *0* non-acceptable gel, *–* no participation

^a^Total percentage score of the maximum score of 15 points

In two of the five gel analysis parameters, only acceptable scores were generated; all participants defined lanes and curves correctly in accordance to the guidelines in all three EQA rounds (Table 7). A few (*n* = 6) non-acceptable scores were obtained by four laboratories (no. 128, 129, 130, and 148) for the three other parameters. Laboratories no. 128 and 130 placed the gel frame incorrectly by including the wells in the first step of the gel analysis procedure, leading to the possibility of incorrect auto-band search and band assignment. Laboratories no. 128, 129, and 148 performed incomplete band assignments by failing to assigning all bands to the profiles of the test strains (non-acceptable score in the parameter band assignment) and/or to the reference profiles (non-acceptable score in the parameter normalization).

**Table 7:** Number of non-acceptable PFGE gel analyses, average score in *Salmonella* EQA by parameters and by year, 2013–2015

Parameter	Condition for excellent score (﻿3)	No. of non-acceptable analyses (%)			Average score		
		2013 *n* = 16	2014 *n* = 17	2015 *n* = 17	2013	2014	2015
Position of frame	Correct placement of frame and gel inverted	1 (6%)	1 (6%)	0	2.6	2.4	2.6
Strips	All lanes correctly defined	0	0	0	2.9	2.3	2.6
Curves	1/3 of the lanes used for averaging curve thickness	0	0	0	2.7	2.7	2.9
Normalization	All bands correctly assigned in all reference lanes	0	2 (11%)	0	2.8	2.5	2.8
Band assignment	Bands assigned correctly according to gel quality	1 (6%)	1 (6%)	0	2.8	2.6	2.6

Scores given according to the BioNumerics Gel Quality Grading Guidelines, [18–20]. Scores of 1: non-acceptable gel, scores of 2 or 3: acceptable

### MLVA

Overall, 15 of the participating NPHRLs (52%) performed MLVA typing; most (12/15) participated in all three EQA rounds (Table 8). The performance level was high, with only 15 (4%) incorrect allelic profiles among 420 profiles reported, and more than half (8/15) of the participants generated 100% correct allelic profiles. Six laboratories (no. 49, 88, 100, 142, 144, and 148) reported incorrect profile(s) in one EQA; half of these were in the first 2013-EQA, and one (no. 77) reported incorrect profiles in two EQA rounds. Four laboratories (no. 49, 142, 144, and 148) improved their level of performance, and two laboratories (no. 77 and 88) showed variable performance level by reporting fewer correct allelic profiles in later EQA rounds compared to previous (i.e., 2013-EQA and 2014-EQA for laboratory no. 77 and 2013-EQA for laboratory no. 88 respectively) (Table 8). Laboratory no. 77 swapped two isolates in the 2015-EQA, and laboratory no. 88 swapped two alleles and failed to read fragment sizes above 509 base pairs on the chromatogram in the 2015-EQA.

**Table 8:** Individual laboratory MLVA result in *Salmonella* EQA rounds by year, 2013–2015

Number of EQAs (no. of participants)	Laboratory number	Number of correct MLVA profiles					
		Test strains (*n* = 10)			Repeat strains (*n* = 3)		
		2013	2014	2015	2013	2014	2015
3 (*n* = 12)	19	10	10	10	3	3	3
	36	10	10	10	3	3	3
	129	10	10	10	3	3	3
	134	10	10	10	3	3	3
	147	10	10	10	3	3	3
	149	10	10	10	3	3	3
	100	10	7	10	3	2	3
	77	10	9	8	3	2	2
	49	9	10	10	3	3	3
	142	9	10	10	3	3	3
	144	8	10	10	3	3	3
	148	10	9	10	3	3	3
2 (*n* = 3)	150	10	10	–	3	3	–
	88	10	–	6	3	–	2
	108	–	10	10	–	3	3

SLVs were accepted as a correct result for five laboratories when evaluating the reported allelic profiles. In the 2013-EQA, a SLV in locus STTR5 (allele 12 changed to 13) of test strain 16 was accepted for four laboratories (no. 49, 77, 148, and 150). Furthermore, a SLV in locus STTR10 (allele 3 changed to 4) of test strain 19 was accepted for laboratory no. 148.

The 2015-EQA distribution included a SLV (test strain 17) of one of the repeat strains (c, Table 2). The new allele was stable during the stability test, and reported by all participants identifying the locus, reporting correct allelic profiles for both strains.

General improvement was observed over the three EQA rounds; three (21%) laboratories generated incorrect profiles in the 2013-EQA, whereas only two (14%) laboratories reported incorrect profiles in the 2015-EQA. In the 2014- and 2015-EQA, all participants used the agreed nomenclature, reporting alleles as true number of repeat units and NA/−2 for absent loci [19, 20].

The 15 incorrect allelic profiles represented 25 allele errors. Twelve of the errors reported were switching of either two isolates or two alleles. Additionally, eight were missing the presence of a locus, four errors were related to reporting incorrect allele number, and one error was reporting the presence of a locus where there was none. No common strain characteristics or allele numbers generated errors, and no laboratory repeated the same error in two rounds.

## Discussion

Definitive characterization of foodborne pathogens by molecular typing is essential for surveillance, outbreak detection, and source identification. The globalization of food product trade implies that multi-country food-associated outbreaks may occur more frequently [22–25]. Cross-border comparison of molecular typing data is dependent on robust and comparable standard typing methods. For *Salmonella*, standard PFGE and MLVA protocols [11, 13–15] and nomenclature have been developed [12, 21]. Laboratory procedures for the methods accepted for EU-wide surveillance are available at the ECDC website (http://ecdc.europa.eu/en/healthtopics/food_and_waterborne_disease/surveillance/Pages/index.aspx). However, the full potential of cross-border surveillance relies on ability of the laboratories to generate high-quality typing data routinely and share data as close to real-time as possible. Therefore, this study addressed the quality of the typing data reported from the European NPHRLs expected to share data in TESSy in order to identify critical parameters for improving surveillance, laboratories in need of technical assistance, and performance development.

PFGE has been the international gold standard for molecular subtyping of *Salmonella*, and almost all (27/29) participating NPHRLs registered to this part of the assessment scheme. The quality of a PFGE gel depends on strict adherence to the standard protocol. Even slight deviations from the procedure may result in reduced quality and comparability. This was reflected in the low number of gels (7%) obtaining a total 100% score. Only three laboratories (10%) managed to produce perfect gels, which highlights the challenges related to this method. However, an excellent gel quality is not a necessity for inter-laboratory comparison of profiles, and only two laboratories were unable to produce profiles of high enough quality to be useful for comparison. To improve the performance of these two laboratories, direct technical assistance or training might be useful, as the detailed feedback on their specific problems through individual evalution reports did not circumvent their low performance.

The comparability of profiles relies primarily on the use of correct running conditions, good quality image acquisition, and distinct bands. Indeed, these gel parameters, “image acquisition and running conditions” and “bands”, generated non-acceptable profiles in all EQA rounds. The additional gel parameters received an acceptable score in all cases and seem to be more robust to deviations.

It is of utmost importance to use the PFGE protocol for the relevant organism; however, some (*n* = 4) laboratories were suspected of applying incorrect running conditions, e.g., the electrophoresis conditions for *E. coli* O157 were used for *Salmonella*. Normalization of bands according to an international standard directly depends on correct standard running conditions and adequate numbers of reference lanes. Thus, deviation from the standard renders the profiles incapable of being compared. Laboratories performing PFGE routinely should be expected to use correct running conditions as detailed in the provided protocols. In addition to the use of correct running conditions, proper image acquisition and distinct bands are essential. Production of clear and distinct bands is highly susceptible to deviations from the protocol, and indeed the parameter “bands” obtained the lowest average score through the three EQA rounds.

Fuzzy and/or thick bands were common causes for non-acceptable and low “band’ scores. In a few cases (4/14 poor band scores), entire lanes were distorted as well. Several factors cause bands to appear fuzzy; poor image capture by improper focusing, use of too narrow wells, and thick gel slices >2 mm. In the specific feedback, laboratories were encouraged to evaluate their in-house procedure systematically to identify even slight deviations from the standard protocol. Low-quality image acquisition highly affects the band quality. The presence of weak bands most likely led several laboratories to increase the exposure time and/or enhance the contrast of the gel image, causing the bands to appear too thick to separate double bands. Thus, laboratories presumably producing a gel of acceptable quality failed to document this due to improper image capture. A thorough protocol on correct image acquisition was distributed to the participants in the 2014- and 2015-EQA rounds, and the quality increased from 2014 to 2015. The combined gel parameter “image acquisition and running conditions” could be separated into two different parameters in future EQA rounds to assess the parameters individually.

It can be speculated that the overall quality of PFGE gels produced in NPHRLs not performing this method routinely would diminish. Thus, the substitution of PFGE with whole genome sequence (WGS) typing methods will reduce gradually the need for PFGE EQA schemes. Future assessment schemes should represent the different methodologies applied in parallel in the EU, including PFGE, MLVA and WGS-based analyses.

Participants could select which parts of the EQA to perform, but although the majority (27/29) of the participating NPHRLs performed PFGE typing, only 19 performed the subsequent profile analysis. Laboratories potentially lack access to the software or lack experience in analysing PFGE profiles. However, PFGE-based analysis requires the capacity to analyse PFGE profiles. The performance level displayed by those laboratories participating in the gel analysis was high and improving during the 3-year period. In the EQA-2015, all laboratories demonstrated the ability to produce an acceptable gel analysis in accordance with the guidelines.

The overall performance level for MLVA typing was high and improving through the EQA rounds; all participants reported 100% correct allelic profiles in at least one EQA round. A low rate of 4% (25/420) of misreported allelic profiles was seen among the 420 profiles reported in the 3 years. These errors could have been avoided by proofreading of the results and by taking into consideration the known characteristics of the individual locus, e.g., allele out of expected range, allele not seen before, or locus always present. Absence of amplification of STTR3 was reported a total of five times by two laboratories, although absence of this locus should be considered highly unlikely. In the MLVA scheme, only absence of the loci STTR6 and STTR10 is likely [5]. Furthermore, two of the four incorrect allele numbers reported for STTR3 (allele 409 and 410) are uncommon alleles. STTR3 is a combined locus of two repeat sizes, consisting of 0–5 27-basepair repeat units and 8–14 33-basepair repeat units. However, not all combinations of these seem to occur [12]. Since the incorrect allele numbers of STTR3 were only reported in two of the ten test strains in one of the three EQA rounds, it was not considered as a general error in the participant’s procedure. Critical evaluation of results and the use of checkpoints to control the quality and to assess if values are within the expected range and is of high importance.

In summary, lack of proofreading contributed considerably to the errors identified in the MLVA typing. Although these errors were few and should be easy to avoid, uploading of incorrect allelic profiles to TESSy can have significant impact on outbreak detection, since MLVA data are not curated. Thus, proofreading by experienced personnel should be done before sharing of data. TESSy could improve by curation of MLVA data, i.e., confirmation when rare alleles occurs.

Recently, a standard operating procedure for MLVA typing of *S*. Enteritidis was published [26], and MLVA data for this serovar are now collected in TESSy. Future EQA rounds will include this method to foster harmonization and ensure data quality in the same way as for *S*. Typhimurium. In Europe, *S*. Enteritidis and *S*.Typhimurium are the two most common serovars reported in humans, and MLVA typing provides high resolution with good epidemiological concordance for both serovars [27]. In comparison to whole genome sequence (WGS) typing methods, MLVA is low-cost, and easier to perform and interpret.

## Conclusion

The EQA schemes strengthened the laboratory network of FWDs at the EU/EEA level as the number of laboratories participated increased, and results improved during the 3-year period. More harmonized and reliable typing methods, particularly molecular typing techniques, allow: (a) effective analysis to detect unusual events, detect uncommon increase of cases and possible national and cross-border outbreaks, (b) increased capacity for further characterization of human isolates, more accurate delineation of outbreaks, and (c) source identification and verification, comparison with data from the food/animals sector.

The participation in the PFGE laboratory part of the assessment was high, whereas the *S.* Typhimurium specific MLVA method received a lower participation. This emphasizes the need for continued assessment of the different methodologies applied in the NPHRLs in Europe, including both classical methods and new WGS-based typing methods, as it should be anticipated that several methods will be used in parallel by the different NPHRLs in a transition period. This poses a challenge for European-wide surveillance, but the historical data on PFGE and MLVA, combined with the possibility of producing WGS-based typing data on different resolution levels, improves the possibility, of to some degree, correlating results obtained by different methods. Currently, a harmonized procedure for WGS data analysis in routine surveillance remains to be developed. Future EQA shemes could foster the development and harmonization of WGS-based surveillance at the EU level; however, in the transition period, PFGE and MLVA will be a valuable tools for comparison.

External quality assessment is a valuable tool for identifying areas for improvement, and strong and improved performance levels were demonstrated through the course of the three EQA rounds. For PFGE, the main gel quality problems were related to image acquisition, running conditions, and indistinct bands. The current EQA scheme have especially supported the European NPHRLs to use the standard running conditions for PFGE, uniform PFGE band assignment procedures, use of MLVA reference strains, as well as uniform MLVA allelic nomenclature. The European NPHRLs’ interest in improving the molecular typing data quality and the participation in EQA schemes is important for the quality of the European-wide surveillance of foodborne infections.
